# Frailty in older people: Rehabilitation Treatment Research Examining Separate Settings (FORTRESS): protocol for a hybrid type II stepped wedge, cluster, randomised trial

**DOI:** 10.1186/s12877-022-03178-1

**Published:** 2022-06-27

**Authors:** Heather Block, Alexandra Annesley, Keri Lockwood, Linda Xu, Ian D. Cameron, Kate Laver, Maria Crotty, Catherine Sherrington, Annette Kifley, Kirsten Howard, Dimity Pond, Tuan A. Nguyen, Susan E. Kurrle

**Affiliations:** 1 Rehabilitation, Palliative and Aged Care Division, Southern Adelaide Local Health Network, Adelaide, Australia; 2 Rehabilitation and Aged Care Services, Northern Sydney Local Health District, Sydney, Australia; 3 Faculty of Medicine and Health, University of Sydney, Sydney, Australia; 4 John Walsh Centre for Rehabilitation Research, Faculty of Medicine and Health, University of Sydney, Sydney, Australia; 5 Flinders Health and Medical Research Institute, College of Medicine and Public Health, Flinders University, Bedford Park, South Australia Australia; 6 The Menzies Centre for Health Policy and Economics, School of Public Health, Faculty of Medicine and Health, University of Sydney, Sydney, Australia; 7 School of Medicine and Public Health, University of Newcastle, Newcastle, Australia; 8 National Ageing Research Institute, Melbourne, Australia; 9 School of Health Sciences, Swinburne University of Technology, Melbourne, Australia; 10 UniSA Clinical and Health Sciences, University of South Australia, Adelaide, Australia

**Keywords:** Frailty, Aged, Implementation, Hospital, Older people, Exercise, Nutrition, Transition, Primary care

## Abstract

**Background:**

Frailty in older people is associated with increased risk of falls, longer length of stay in hospital, increased risk of institutionalisation and death. Frailty can be measured using validated tools. Multi-component frailty interventions are recommended in clinical practice guidelines but are not routinely implemented in clinical practice.

**Methods:**

The Frailty in Older people: Rehabilitation, Treatment, Research Examining Separate Settings (FORTRESS) trial is a multisite, hybrid type II, stepped wedge, cluster, randomised trial with blinded assessment and intention-to-treat analysis being conducted in Australia. The study aims to determine the effectiveness and cost-effectiveness of an embedded individualised multicomponent frailty intervention (commencing in hospital and continuing in the community) on readmissions, frailty and quality of life when compared with usual care. Frail older people admitted to study wards with no significant cognitive impairment, who are expected to return home after discharge, will be eligible to participate. Participants will receive extra sessions of physiotherapy, pharmacy, and dietetics during their admission. A Community Implementation Facilitator will coordinate implementation of the frailty management strategies and primary network liaison. The primary outcome is number of days of non-elective hospital readmissions during 12 month follow-up period. Secondary outcomes include frailty status measured using the FRAIL scale; quality of life measured using the EQ-5D-5L; and time-to-event for readmission and readmission rates. The total cost of delivering the intervention will be assessed, and cost-effectiveness analyses will be conducted. Economic evaluation will include analyses for health outcomes measured in terms of the main clinical outcomes. Implementation outcomes will be collected as part of a process evaluation. Recruitment commenced in 2020 and we are aiming to recruit 732 participants over the three-year duration of the study.

**Discussion:**

This study will reveal whether intervening with frail older people to address factors contributing to frailty can reduce hospital readmissions and improve frailty status and quality of life. If the FORTRESS intervention provides a clinically significant and cost-effective result, it will demonstrate an improved approach to treating frail patients, both in hospital and when they return home.

**Trial Registration:**

Australian New Zealand Clinical Trials Registry (ANZCTR): ACTRN12620000760976p. ANZCTR registered 24 July 2020.

**Supplementary Information:**

The online version contains supplementary material available at 10.1186/s12877-022-03178-1.

## Background

Frailty is a clinical state with multiple causes and contributors in which there is an increase in an older person’s vulnerability for developing increased dependency and/or mortality when exposed to a stressor [1]. The likelihood of being frail increases with age, and frailty is associated with increased risk of falls, longer length of hospital stay, and increased risk of institutionalisation and death [2]. A recent Australian study estimated that 21% of community-dwelling people aged 65 years and over are frail with a higher proportion of women being frail. Approximately 48% of this older population were found to be prefrail [3]. Within hospital settings, Richards et al., [4] reported 49% of adult inpatients were being classified as frail. Prior studies have reported frailty prevalence rates of between 27 and 80% in hospitalised older patients [5, 6].

Frailty is measurable using a range of approaches and validated tools. Two major approaches to identifying frailty are the frailty phenotype and the accumulated deficits model. Frailty phenotype defines frailty as having three or more of: unexplained weight loss, low grip strength, slow walking speed, low physical activity, and self-reported exhaustion [7]. Validated frailty phenotype tools include questionnaires such as the FRAIL Scale [8], and performance-based measures such as the Cardiovascular Health Study (CHS) (often termed “Fried”) Frailty Phenotype [7]. The accumulated deficits model counts diseases, conditions and comorbidities [9, 10]. Accumulation deficit tools measure the number of medical conditions, psychological conditions, physical and cognitive function, and social factors in the Frailty Index [10], or the Clinical Frailty Scale [11] derived from it. Both of these approaches have been shown to be useful for identifying people at high risk of adverse events as a result of their frailty [12]. Whilst many frailty screening instruments are useful in identifying frailty, they do not necessarily direct clinicians to appropriate interventions [13]. The frailty phenotype approach (using the FRAIL Scale or the CHS Frailty phenotype) can give the clinician clear direction for interventions through focussing on the factors that have been identified by the instrument as contributing to frailty. There are generally considered to be four evidence-based areas of intervention for older people with frailty. These are physical exercise, nutritional interventions, multicomponent interventions and individualised care from staff with expertise in geriatric medicine [13–15].

The Asia Pacific Clinical Practice Guidelines for the Management of Frailty [16] provide evidence-based recommendations, including (i) using validated tools to identify frailty, (ii) prescription of physical activity incorporating resistance training, (iii) address polypharmacy, (iv) provide nutritional support, (v) screen for reversible causes of fatigue, and (vi) Vitamin D supplementation. However, guidelines do not implement themselves [17] and there is a need to increase the use of comprehensive, integrated care to better manage patients with frailty, to improve treatment adherence and increase healthy ageing among the older population [18].

The Frailty in Older people: Rehabilitation, Treatment, Research Examining Separate Settings (FORTRESS) study will address this gap through evaluating use of a validated frailty screening tool to guide implementation of an evidence-informed intervention for frailty in the acute hospital setting, with follow up in the community and general practice setting. This pragmatic implementation trial is required to understand effectiveness, cost-effectiveness, adoption, acceptability and adherence of evidence-based frailty identification and management [19].

## Methods

### Design

**Fig. 1:**
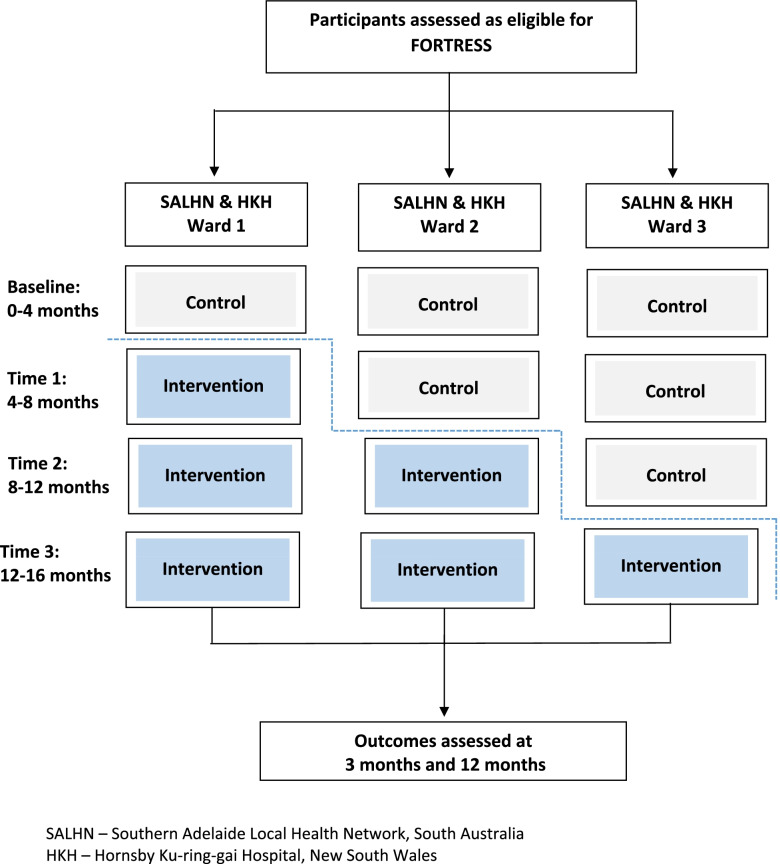
Overview of the FORTRESS stepped wedge design Participants and sites. SALHN – Southern Adelaide Local Health Network, South Australia. HKH – Hornsby Ku-ring-gai Hospital, New South Wales

The FORTRESS trial is a multi-site hybrid type II stepped wedge, cluster randomised trial to be conducted over three years (see Fig. 1). This study protocol is reported in accordance to the Standard Protocol Items: Recommendations for Intervention Trials (SPIRIT) checklist available as supplementary file. The hybrid type II trial approach [20] allows testing of the implementation strategy (Implementation Facilitation [21]) and testing the outcomes of the intervention (screening for frailty and use of appropriate interventions).

The FORTRESS trial builds on previous research conducted by members of the research team. Cameron et al., [22] evaluated the efficacy of a multicomponent interdisciplinary intervention with community dwelling older people after completion of their contact with health services in the Frailty Intervention Trial (FIT). Interventions included multiple sessions of physiotherapy and dietetics, with limited comprehensive geriatric assessment over a period of 12 months. Results of FIT included reduced frailty, improved physical performance, and increased degree of independence for community dwelling older people [22, 23]. Economic evaluation revealed that in very frail older people residing in the community, the multicomponent interdisciplinary intervention was both more effective and less costly than usual care [23, 24].

The protocol for this study has been approved by the Northern Sydney Local Health District Human Research Ethics Committees and is registered at www.ANZCTR.org.au (ACTRN12620000760976p).

The FORTRESS trial will be conducted across six inpatient wards at two different states (Hornsby Ku-ring-gai Hospital, Northern Sydney Local Health District in New South Wales, and Southern Adelaide Local Health Network in South Australia). Older people will be included if they are aged 75 years or older; admitted to the six wards included within the participating local health districts; assessed as frail (score of 3 or above out of 5 on the FRAIL scale [8]); living at home in the community; and score 24 or above out of 30 on the Mini Mental State Exam [25]. People will be excluded if they are admitted to hospital with a new diagnosis of stroke (people with acute stroke are admitted to specialised units in both participating hospitals), have an illness likely to be associated with a life expectancy of < 12 months (assessed on the modified version Illness Severity Rating [20]); are unable to cooperate with the intervention program; were not mobile prior to admission; or reside in a residential aged care home. 

As the trial involves implementation of best-practice guideline recommendations [16] we will use an opt-out approach to participant consent. If people elect to opt-out of the study, they will receive usual care, but will not receive the frailty specific interventions while in hospital and they will not be followed up in the community once discharged.

Partnership with general practices will involve provision of training webinars to the GPs and practice nurses, and GP practice visits for education sessions to practices within the local health networks involved in FORTRESS trial. Education sessions entail primary care frailty management with training on frailty screening, frailty management plans. Within Northern Sydney Health Network, a small group of general practices will be engaged at a one-to-one level to understand the frailty screening process and identify service gaps which can assist in developing co-commissioning strategies. These general practices will provide monthly reporting and feedback for a period of 12 months.

### Personnel

Two study coordinators (one in NSW and one in SA) will be responsible for project management. Allied health professionals (Implementation clinician, Implementation physiotherapist/Exercise Physiologist, Implementation Pharmacist, Implementation Dietitian) will provide extra sessions of physiotherapy, pharmacy, and dietetics for each ward while the person is an inpatient. A therapist will act as a Community Implementation Facilitator will work with people upon discharge from hospital to assist with implementation of the frailty management strategies and primary network liaison. 

### Intervention

Patients admitted to the wards taking part in this study are screened for eligibility. Baseline frailty is measured using the FRAIL scale [8] and a score of 3 and above indicates frailty. If the person has been admitted to a ward during the control phase of the research study, they will receive usual care as provided by the ward staff and their general practitioner and other health service providers. Participants admitted to the ward during the intervention phase will receive an individualised intervention designed to treat the identified components of frailty, along with the community implementation visit(s), telephone calls, and support from their general practitioner (GP) once discharged. Additional file 1 outlines the TIDier framework [26] describing the FORTRESS intervention. Intervention is divided into two phases:

#### The Hospital Intervention

Intervention participants will receive referrals for physiotherapy, dietitian and pharmacy review. A tailored Frailty Management Plan will be developed by the multidisciplinary team and documented in the electronic medical record. A tailored exercise program involving resistive training prescribed by the physiotherapist and/or exercise physiologist. Malnutrition screening, nutrition education and advice will be provided by the dietitian. When indicated, the pharmacist will undertake medication history and review at hospital admission and medication reconciliation at discharge, provide education on medication changes and prepare a medication management plan. Indicators for pharmacy review include those experiencing frailty side effects of medication and polypharmacy. The pharmacist will document in the Frailty Management Plan any changes in medication regimen in hospital and the reasons for change; and recommend referral for a Home Medicines Review to be completed within 3 months of discharge. Other frailty interventions such as commencing Vitamin D supplementation may occur when indicated by the medical team or pharmacist. Prior to discharge from hospital, the Frailty Management Plan will be discussed with the participant and/or their significant other who will also receive a copy. Any nutrition and/or exercise recommendations will be detailed and provided in a handout for participants. A copy of the Frailty Management Plan will be sent via fax or secured electronic messaging to the participant’s GP along with all other relevant discharge information.

#### The Community Intervention

A health professional employed as a Community Implementation Facilitator will review the participant’s motivation and adherence to the Frailty Management Plan recommendations after discharge from hospital and will liaise with the person’s general practice to facilitate ongoing frailty management. The participant will receive a home visit from the Community Implementation Facilitator within a week of discharge, and 2–4 telephone calls in the subsequent 4–6 weeks post discharge. The role of the visits and telephone calls are to support adherence to frailty management plan recommendations, including:

- Clarify the purposes of the study and answer any questions that the participant or their carer/significant other may have, that have arisen since the participant’s discharge
- Ensure study participants understand and can perform the prescribed exercises in their home environment
- Encourage study participants to continue with the nutrition interventions at home
- Assist/encourage participants to make an appointment to see their GP as soon as practicable following discharge
- Contact the GP or Practice Nurse with suggested ongoing management of the participant’s Frailty Management Plan

The pharmacist will telephone participants post-discharge to provide detailed education on current medications and recent hospital medication changes. The pharmacy telephone call will entail education to the participant about their medications and encourage them to adhere to the medication management plan prepared in hospital. The Home Medicines Review will be recommended to be initiated by the GP to ensure any medication changes will be implemented by the GP. The Home Medicines Review report will be sent to the participant’s GP, and the study coordinator where available.

### Outcome measurements

#### Primary outcome

The primary analysis will compare length in days of non-elective hospital admissions after the index admission during 12 months of follow up between intervention and control conditions. Electronic medical records for each participant will be reviewed at 3 months and 12 months for non-elective public readmissions at hospitals within the included local health district. Private hospital readmissions are not available on the public electronic medical records, therefore will be determined by participant self-report to a blinded outcome assessor at 3 months and 12 months. Reason for readmission will also be gained from electronic medical record and participant self-report, to determine Diagnostic Related Groups (DRGs).

#### Secondary outcomes

1. Frailty status at baseline, 3 months and 12 months measured using the FRAIL Scale [8].
2. Quality of life at baseline, 3 months and 12 months measured using the EQ-5D-5L [27].
3. Time-to-event of readmission, and readmission rates at 3 months and 12 months.

Baseline measures are assessed by the study Implementation Clinician on the ward at admission. Follow-up outcome assessment will take place via telephone at 3 and 12 months by a health professional masked to group allocation. Each assessment will include administration of the FRAIL Scale to measure frailty and EQ-5D-5L to measure quality of life. Data regarding overnight non-elective hospital readmissions will be obtained from participant self-report, and from public hospital electronic medical records for hospital readmissions.

#### Additional measures

Participants will be asked to self-report their weight and current medications at 3- and 12-month outcome assessments. Current medications will be cross-checked through participant’s pharmacists and Home Medicine Review report where available. Covariables to be considered will include age, sex, degree of frailty at index admission, number of hospitalisations in the preceding 12 months, cognition (MMSE score), and primary admission diagnosis for the index admission and for readmissions.

**Fig. 2:**
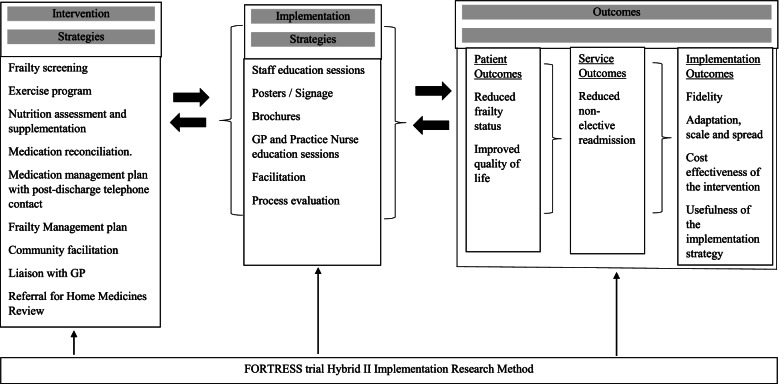
Conceptual model of hybrid II implementation research for FORTRESS trial

The hybrid nature of this trial allows understanding of both effectiveness and implementation. Figure 2 describes the conceptual model of evaluation [28]. A process evaluation will be conducted concurrently to describe the adaptation, scale and spread of FORTRESS intervention and details will be described in a separate paper. Details are available through the trial registry.

### Sample size

The projected enrolment is approximately 732 participants from the six wards over 16 months. This sample size enables detection of a mean difference of 35 days versus 50 days in total length of hospital stay at readmission after the index admission during 12 months of follow up, with 80% power at significance level 5%, allowing for 10% mortality, 10% dropout, and a design effect of 1.75 for clustering by wards.

### Statistical analysis

The primary analysis will compare length of stay in hospital at readmission after the index hospital admission during 12 months of follow up between intervention and control conditions, using generalised linear mixed models with a negative binomial distribution, accounting for clustering by wards and adjusting for credible confounders to further control baseline imbalance. The approach will be intention to treat.

Secondary analyses will compare time to event of readmission between intervention and control conditions using survival analysis, and frailty status, quality of life outcomes and readmission rates using linear mixed models, accounting for clustering by ward and adjusting for credible confounders.

Covariables to be considered in these analyses will include age, sex, degree of frailty at index admission, number of hospitalisations in the preceding 12 months, cognition (MMSE score), and primary admission diagnosis for the index admission and for readmissions. SAS and R statistical software will be used for analysis.

### Randomisation procedure

Transition to the intervention phase will be randomised by ward with stratification by site and using minimisation with a random component to optimise baseline and sequential balance. To do this the minimisation algorithm will use anticipated ward characteristics including the type of ward, typical length of stay, high or low tendency to discharge to a day rehabilitation ward rather than home and, where possible, typical readmission rates at that ward.

### Implementation evaluation

Implementation strategies were informed based on consideration of the Consolidated Framework for Implementation Research (CFIR). The CFIR consists of 40 constructs organised into 5 domains: Intervention Characteristics, Outer Setting, Inner Setting, Characteristics of Individuals, and Process [21]. Implementation facilitation on the wards (during the intervention phase) include staff (employed by the research team) facilitating frailty screening and frailty management plans, multiple education sessions for multi-disciplinary team members, signage and documentation (journey board), and posters within the ward to increase awareness. Implementation facilitation has been shown to enhance uptake and sustainability of evidence based practice [29]. Implementation Facilitation will be evaluated through mixed methods including interviews and assessing levels of engagement. Fidelity to the intervention will be measured by the Community Implementation Facilitator reporting adherence to frailty recommendations and motivation of participants in a checklist. Interviews with hospital staff, study participants and their carers will be conducted following hospital discharge. A process evaluation of implementation strategies will be conducted concurrently to FORTRESS intervention with details to be described in a separate paper.

### Economic analysis

The total cost of delivering the intervention will be assessed, and cost-effectiveness analyses will be conducted, according to the principles for conducting economic evaluation alongside stepped wedge trials [30]. The economic evaluation will take the perspective of the health care funder and will include analyses for health outcomes measured in terms of the main clinical outcomes, including cost per extra person avoiding readmission, cost per extra person avoiding frailty, and cost per quality adjusted life years (QALY) gained based on the EQ-5D-5L. The study will collect data on the cost to deliver the intervention (including staff costs, training, capital costs and consumables), and inpatient hospital admissions, based on DRG-related costs. Using the mean costs in each trial arm, and the mean health outcomes in each arm, the incremental cost per extra person avoiding frailty, per extra person avoiding re-admission and per QALY gained of the intervention group compared with control group will be calculated; results will be plotted on a cost-effectiveness plane. Bootstrapping will be used to estimate a distribution around costs and health outcomes, and to calculate the confidence intervals around the incremental cost-effectiveness ratios. One-way sensitivity analysis will be conducted around key variables. A probabilistic sensitivity analysis will be conducted to estimate the joint uncertainty in all parameters, and a cost-effectiveness acceptability curve (CEAC) will be plotted.

### Data management

All project data collected from participants and the electronic medical record will be entered in a REDCap (Research Electronic Data Capture) which only the investigators and members of the project team have access to. Data will be checked and cleaned prior to analysis. A data monitoring committee was not established as this trial involves implementing best practice (guideline recommendations). Any serious adverse events related to the intervention (such as a fall on a home visit while practising exercises) will be reported to the ethics committee.

### Time frame

Recruitment will commence in December 2020. Follow up assessment is expected to conclude in December 2023.

## Discussion

Frailty is associated with poorer outcomes for individuals and prevalence is likely to increase due to an ageing population. There is evidence that intervention can effectively reverse frailty and Asia Pacific Guidelines recommend screening and multidisciplinary intervention [16, 22, 23]. This study investigates the efficacy of implementing frailty guidelines for hospitalised older adults and explores the process of implementation in this setting. The results will provide important information which can be used by health care policy makers, managers and clinicians worldwide.

Frail older people with chronic and complex health conditions have high re-admission rates with approximately 25% of older people being readmitted within three months of hospital discharge [31]. Continuity and integration of care following discharge from the acute setting is consistently recommended but difficult to achieve given the current divide between funding for acute and primary care settings. The FORTRESS study aims to work across settings to address frailty identified in a hospital environment. Evidence for cost-effectiveness of preventive, integrated care is generally limited due to a wide variety in study populations, interventions and evaluations [32]. However, previous studies have demonstrated that a multicomponent interdisciplinary frailty intervention is both more effective and less costly than usual care [24]. The FORTRESS study aims to demonstrate that the cost of delivering the intervention will be significantly outweighed by the cost of hospital re-admissions.

A strength of the FORTRESS study is not only the utility of evidence-based guidelines but how they complement a robust and validated screening tool to provide a comprehensive management plan that is readily transferable to clinical practice in both acute and community aged care settings. Further strengths of the FORTRESS study lie in the Frailty Management Plan, designed in hospital based on this frailty phenotype approach and continued at home with the support of the participants’ General Practitioner and a Community Implementation Facilitator. Designing the plan in hospital allows for a timely and complete multi-disciplinary approach, building a strong management foundation that can be continued once the participant has returned home, with strong evidence to support this approach in terms of surviving the acute admission as well as continuing to live in the community with less cognitive decline [33]. A potential weakness of the study design is that it requires the input of multiple health professionals who are providing routine care to the person with frailty. As a result, it may be difficult to achieve adequate adherence with the intervention.

If the FORTRESS intervention demonstrates a clinically significant effect and cost-effectiveness, it will provide an improved approach to treating frail patients, both in hospital and when they return home. An evaluation of how well the service can be provided and how well it is accepted by participants will further inform how this model can best be translated into usual practice.

## Supplementary Information

**Additional file 1.** FORTRESS SPIRIT Checklist: Recommended items to address in a clinical trial protocol and related documents.

**Additional file 2.** SPIRIT 2013 Checklist: Recommended items to address in a clinical trial protocol and related documents.

## Data Availability

Not applicable.

## Abbreviations

ANZCTR: Australian New Zealand Clinical Trials Registry
CEAC: Cost-effectiveness Acceptability Curve
CFIR: Consolidated Framework for Implementation Research
DRG: Diagnosis Related Groups
FORTRESS: Frailty in Older People: Rehabilitation Treatment Research Examining Separate Settings
GP: General Practitioner
HKH: Hornsby Ku-Ring-Gai Hospital
NSLHD: Northern Sydney Local Health District
NSW: New South Wales
QALY: Quality Adjusted Life Years
SALHN: Southern Adelaide Local Health Network
SA: South Australia
