# Supplementary material for: Frailty in older people: Rehabilitation Treatment Research Examining Separate Settings (FORTRESS): protocol for a hybrid type II stepped wedge, cluster, randomised trial
Source: BMC Geriatr. 2022 Jun 27;22:527. doi: 10.1186/s12877-022-03178-1 (PMC9235164; doi:10.1186/s12877-022-03178-1)
Supplement: Supplementary file 1 — Additional file 1. FORTRESS SPIRIT Checklist: Recommended items to address in a clinical trial protocol and related documents. [file 12877_2022_3178_MOESM1_ESM.docx]

Additional file 1. FORTRESS TIDier framework

| **Item number** | **Item description** |
| --- | --- |
|  | **BRIEF NAME** |
| **1.** | FORTRESS program incorporating multicomponent frailty management interventions |
|  | **WHY** |
| **2.** | There is a need to increase the use of comprehensive, integrated care to better manage patients with frailty, to improve treatment adherence and increase healthy ageing among the older population. FORTRESS intervention implements clinical practice guideline recommendations for the management of frailty including physical activity, address polypharmacy, nutritional support. |
|  | **WHAT** |
| **3.** | Participants receive a tailored Frailty Management Plan to treat identified components of frailty. The Frailty Management Plan includes:   - Tailored exercise program incorporating physical activity training prescribed by a physiotherapist and/or exercise physiologist. Exercise progression, repetition, intensity, dose varies depending on Physiotherapist’s and/or Exercise physiologist’s prescription. Exercise handouts provided - Malnutrition screening, nutrition education and advice provided by a Dietitian. Nutrition handout provided as indicated - Review and reconciliation of medications, education on medicines and medication management plan provided by a pharmacist. Recommendation for referral for Home Medicines Review as indicated - Other frailty recommendations such as Vitamin D supplementation or Geriatrician review may occur as indicated.   Community Implementation Facilitator will review the participant’s motivation and adherence to the Frailty Management Plan including:   - progression of exercise program - Provide education and clarify purpose of intervention - Encouragement to continue recommendations on Frailty Management Plan - Encourage participants to make GP appointment - Verbal contact with GP or practice nurse for ongoing Frailty Management Plan |
| **4.** | Procedures:   - Eligible participants screened for frailty using FRAIL scale - Participants receive additional sessions of physiotherapy and/or exercise physiology, pharmacy and dietetics during their inpatient admission - Frailty Management Plan developed by multi-disciplinary team and documented in the electronic medical record - Frailty Management Plan discussed with participant and provided to participant prior to discharge. Any nutrition or exercise recommendations are detailed in a handout - Frailty Management Plan is sent to participant’s GP along with all other discharge information - Community Implementation Facilitator visits participant in their home after discharge and makes 2-4 telephone calls to the participant in the subsequent 4-6 weeks. Community Implementation Facilitator contacts the participant to support their adherence to frailty management recommendations. - Pharmacy phone call to participants after discharge to provide education on medications and support adherence to medication management plan. - Community Implementation Facilitator contacts participant’s GP and/or practice nurse for ongoing management of the participant’s frailty management plan. |
|  | **WHO PROVIDED** |
| **5.** | Intervention provided by trained Allied Health therapists:   - Implementation clinician (Allied Health Assistant and/or Exercise Physiologist) - Implementation Physiotherapist - Implementation Pharmacist - Implementation Dietitian - Community Implementation Facilitator (trained clinician) |
|  | **HOW** |
| **6.** | - Face to face individual sessions with physiotherapist, and or Implementation clinician, pharmacist and dietitian during inpatient admission - Face to face visit with Community Implementation Facilitator at home after discharge - Telephone contact with Community Implementation Facilitator at subsequent contacts - Telephone call from pharmacist - Community Implementation Facilitator makes telephone call to GP and/or practice nurse |
|  | **WHERE** |
| **7.** | - Inpatient sessions with therapists occur at the hospital ward, with adequate space for therapy and client flow. - Community Implementation Facilitator visits the participant in their home with adequate space for exercises and discussion - Subsequent contact with Community Implementation Facilitator occurs via telephone |
|  | **WHEN and HOW MUCH** |
| **8.** | - Additional session of physiotherapy/exercise physiology, pharmacy, dietetics on top of usual care provided during participant’s inpatient admission. - Duration, dose and intensity of exercise varied depending on prescription by physiotherapist/exercise physiologist - Community Implementation Facilitator home visit post-discharge, then 2-4 telephone calls to the participant in the subsequent 4-6 weeks. - Community Implementation Facilitator home visit up to 60 minutes duration and telephone calls up to 20 minutes duration - Telephone calls to GP and/or practice nurse varies as required |
|  | **TAILORING** |
| **9.** | Frailty Management plans included tailored recommendations based on participant’s components of frailty. Recommendations are varied or adapted as prescribed by allied health therapists. |
|  | **ACTUAL** |
| **11.** | Participants adherence to Frailty Management Plan recommendations is assessed by the Community Implementation Facilitator using a fidelity form |
